# Gallbladder containing incisional hernia in an elderly woman

**DOI:** 10.1002/ccr3.7240

**Published:** 2023-04-22

**Authors:** Jerapas Thongpiya, Pitchaporn Yingchoncharoen, Marawan Elmassry, James A. Tarbox, Mahmoud Abdelnabi

**Affiliations:** ^1^ Internal Medicine Department Texas Tech University Health Science Center Lubbock Texas USA

**Keywords:** abdominal pain, gallbladder, incarceration, incisional hernia, strangulation

## Abstract

Gallbladder herniation is a rare phenomenon with risk factors of being female, older age, and previous history of hernias. Imaging modalities can confirm the diagnosis. Cholecystectomy and hernia repair to prevent strangulation may be warranted.

## Abstract 2

Gallbladder herniation is a rare phenomenon with risk factors being female, older age, and history of hernias. Imaging modalities can confirm the diagnosis showing gallbladder absence from its anatomical location and thickened wall. Cholecystectomy and hernia repair may be warranted with a favorable prognosis in cases of incarceration or strangulation.

## 1 CASE PRESENTATION

**FIGURE 1:**
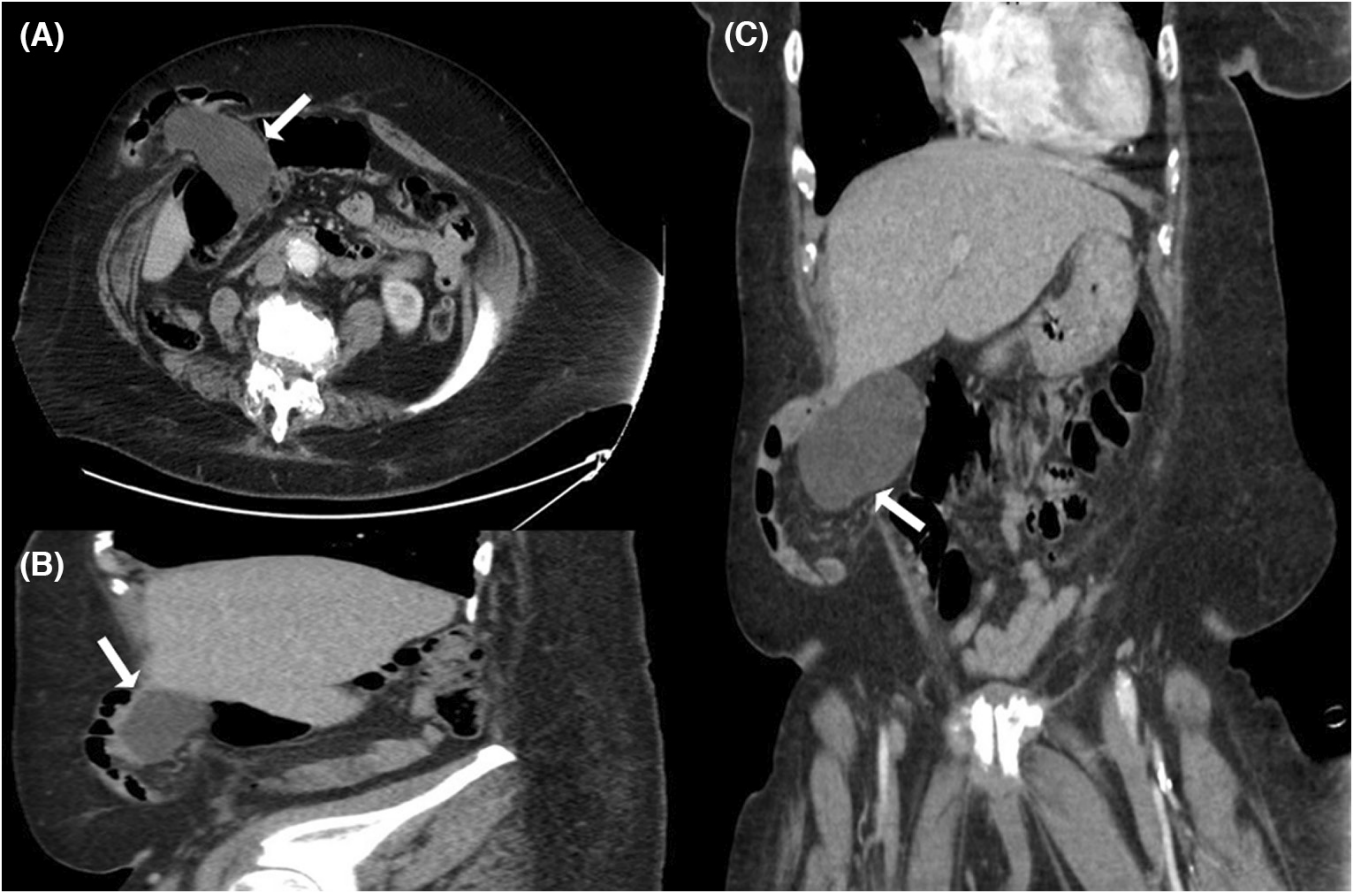
(Panel A–C): Axial, sagittal, and coronal computed tomography (CT) images of the abdomen and pelvis showing a right lower quadrant ventral hernia containing non‐obstructed, non‐distended small intestinal loops and the dome of a distended gallbladder.

A woman in her 70s, with a past medical history of chronic obstructive pulmonary disease on home oxygen supplement 4 liters/minute through a nasal cannula, type 2 diabetes mellitus, hypertension, heart failure with preserved ejection fraction, generalized anxiety disorder, history of clear cell renal cell carcinoma (RCC) status post nephrectomy through an anterior flank incision, right breast invasive ductal carcinoma pending cryoablation therapy, history of provoked subsegmental pulmonary embolism on rivaroxaban, and class III obesity with a BMI of 44 kg/m^2^, presented complaining of a 3‐day history of right‐sided abdominal pain associated with loss of appetite; however, she denied any nausea, vomiting, fever, chills, or constipation. On examination, her vital signs were unremarkable, and her abdominal examination showed a right lower quadrant reducible hernia with mild tenderness without guarding or rebound tenderness. Laboratory workup including complete blood count, comprehensive metabolic panel, and lipase level was unremarkable except for chronic normocytic hypochromic anemia with hemoglobin of 9 gm/dL. Abdominal computed tomography (CT) scan showed a right lower quadrant ventral hernia containing non‐obstructed, non‐distended small intestinal loops, the dome of a distended gallbladder with multiple gallstones. (Figure 1). However, after surgical evaluation, her hernia was deemed non‐incarcerated, so no acute surgical intervention was warranted at this time. After patient counseling due to her multiple comorbidities and high surgical risk, conservative management was considered by avoidance of conditions that can increase intrabdominal pressure such as constipation, pain control, and abdominal wall support using binders with close follow‐up at outpatient settings.

To date, only a few case reports of gallbladder herniation have been published in the literature. The types of gallbladder herniation are variable including internally through the foramen of Winslow, parastomal, or epigastric, incisional, or spontaneous ventral into subcutaneous tissue. Risk factors are being female, older age, and previous history of hernia.^1^ Pathophysiology can be explained by gallbladder mesentery elongation with longevity, anterior abdominal wall weakness, and preexisting hernias.^1^ Patients present with an acute or subacute commonly painful irreducible hernia. Vomiting and bowel symptoms are usually absent if there is no associated bowel obstruction. Inflammatory markers and white cell count are elevated in cases of strangulation or incarceration, but liver function tests are usually normal as no biliary obstruction is present.^1^, ^2^ CT scan is the imaging modality of choice for this diagnosis that can show the absence of gallbladder from its anatomical location and wall thickening without oral contrast filling; it can be also detected via abdominal ultrasonography which may demonstrate communication between the gallbladder and the abnormal cystic mass.^1^, ^2^ Gallbladder herniations through specifically incisional hernia have also been reported and were found to be strangulated requiring surgical intervention, given its narrow neck. Treatment is determined by the presence of incarceration and strangulation, and if present, cholecystectomy and hernia repair with mesh to prevent future herniation may be warranted as attempts to reduce the hernia might result in gall bladder rupture with subsequent peritoneal contamination.^2^, ^3^ Prognosis is favorable in operated cases but remains unknown for non‐surgical options, given the small number of cases reported.

## AUTHOR CONTRIBUTIONS

**Jerapas Thongpiya:** Writing – original draft; writing – review and editing. **Pitchaporn Yingchoncharoen:** Writing – original draft; writing – review and editing. **Marawan Elmassry:** Writing – original draft; writing – review and editing. **James A. Tarbox:** Writing – original draft; writing – review and editing. **Mahmoud Abdelnabi:** Writing – original draft; writing – review and editing.

## FUNDING INFORMATION

No funding was received.

## CONFLICT OF INTEREST

None declared.

## CONSENT

Written informed consent was obtained from the patient to publish this report in accordance with the journal's patient consent policy.

## Data Availability

The data are available for sharing.
